# Eye-tracking en masse: Group user studies, lab infrastructure, and practices

**DOI:** 10.16910/jemr.11.3.6

**Published:** 2018-08-20

**Authors:** Maria Bielikova, Martin Konopka, Jakub Simko, Robert Moro, Jozef Tvarozek, Patrik Hlavac, Eduard Kuric

**Affiliations:** User Experience and Interaction Research Center, Slovak University of Technology in Bratislava; {maria.bielikova, martin_konopka, jakub.simko, robert.moro, jozef.tvarozek, patrik.hlavac, eduard.kuric}@stuba.sk , www.uxi.sk

**Keywords:** Group eye-tracking, eye tracking, gaze, infrastructure, group user study, user experience, usability, reading, interaction

## Abstract

The costs of eye-tracking technologies steadily decrease. This allows research institutions to obtain multiple eye-tracking devices. Already, several multiple eye-tracker laboratories have been established. Researchers begin to recognize the subfield of group eye-tracking. In comparison to the single-participant eye-tracking, group eye-tracking brings new tech-nical and methodological challenges. Solutions to these challenges are far from being established within the research community. In this paper, we present the Group Studies system, which manages the infrastructure of the group eye-tracking laboratory at the User Experience and Interaction Research Center (UXI) at the Slovak University of Technology in Bratislava. We discuss the functional and architectural characteristics of the system. Furthermore, we illustrate our infrastructure with one of our past studies. With this paper, we also publish the source code and the documentation of our system to be re-used.

## Motivation for group eye-tracking

Nowadays, technological advances and vendor competition are steadily lowering the price of the eye-tracking technology. Research institutions can buy more eye-tracking equipment, i.e., more individual eye-tracking stations (12). So much so, the number of eye-trackers can easily surpass the number of rooms available to house them, or the number of personnel available (and able) to use them in a “traditional” study setup. By a traditional setup, we mean studies, in which the participants work one-at-a-time on a single eye-tracking station. In such setup, the study moderators manage the participants one-to-one. As the prices go lower, an institution may want to furnish couple more “traditional setup” eye-tracking labs. This way, however, the low prices of eye-tracking technology cannot really be exploited, because the personnel and overhead costs would need to scale as well.

It may, however, make sense to arrange multiple eye-trackers in a different setup. Multiple eye-tracking stations (e.g., 20 PCs) can be placed together into a *group eye-tracking room*. In such physical setup, studies will no longer have the *one-to-one,* but *one-to-many* design. In such design, the moderator can (at the same time) manage multiple participants working in parallel.

A group eye-tracking setup requires a special infrastructure. The system must provide means to design the experiments, effectively distribute the scenarios to workstations, orchestrate the work and collect the recorded data.

To this day, laboratories with the group eye-tracking setups have existed for some years in several (but not many) institutions worldwide (14, 2, 8, 6, 19). Software solutions with features allowing the control of multiple eye-trackers also started to emerge (12). However, as a discipline, the group eye-tracking is not yet well described, discussed and methodologically established. Only recently, fora dedicated to this field started to emerge. And, seasoned infrastructural solutions are not yet available.

### Contribution of this paper

With this paper, we aim to contribute to the forming field of group eye-tracking. We present the *infrastructure of our group eye-tracking laboratory*, which we developed at the User Experience and Interaction Research Center (UXI) of the Slovak University of Technology in Bratislava.

Our system, called *Group Studies,* places all the eye-tracking stations under one umbrella to be easily controlled. In this paper, we discuss various aspects of this system, mostly through a functional and architectural perspective. We put a strong emphasis on flexibility of the study design process, extensibility and integration of our system to other applications. To better illustrate the potential of our infrastructure, this paper also presents an example study from the domain of programmer eye-tracking.

With this paper, we also publish the source code of our infrastructure along with the necessary technical documentation. Our solution can thus be used by any individual or institution wishing to use the group eye-tracking.

## Background

In comparison with the traditional setup, *group eye-tracking* has several advantages, but also limitations and challenges. Depending on the study requirements, the trade-off between the pros and cons can, in many cases, play in favor of the group setup.

The advantages and benefits of the group eye-tracking include:

1. *Time and effort savings*. If the study participants work in parallel, the total duration of the experiment sessions can be radically cut down. Also, the effort needed to moderate the sessions scales down too (e.g., a couple of moderators to tens of participants). This shortens the studies, which rely on an automated quantitative evaluation. Naturally, if there is a need for manual evaluation (coding) of the recorded sessions, the group setup is only little different to the single device setup – both require the human labor.
2. *Move towards uniform experiment conditions*. The group studies make participants to work at the same time, at the same place and listen tothe same instructions. This lowers the risk of biases caused by uncontrolled environment variables.
3. *Possibilities for collaborative scenarios*. Using multiple eye-tracking stations at once opens a completely new domain of studies, where interaction of participants is involved. Examples already exist, e.g., in collaborative gaming, learning or search (1, 13, 18).

The limitations and challenges of the group eye-tracking include:

1. *Need for a non-trivial infrastructure*, which must provide means for a central control of the study process and enable integration with the experimental and analytic applications, required for the study. Addressing this concern is a primary contribution of this paper.
2. *Study organization issues.* In group studies, we have lesser control over the individual participants, fewer instructing options, tighter scheduling, etc.
3. *Data quality issues*. The lesser control over the participants throughout the experiment may lessen the quality of the acquired eye-tracking data.
4. *Potential interactions between the participants*. Some studies suggest, that the very presence of other participants may influence the outcome of certain metrics (16). The participants may disturb each other, for example by noise. Therefore, certain types of experiments may not be possible (e.g., when we need the participants to express themselves verbally during a think-aloud protocol).

Today, the group eye-tracking requires a custom software and hardware infrastructure. The available eye-tracking software tools (e.g., Tobii Studio, SMI Experiment Center, OGama) are suited for single-user experiments and are generally inadequate for the group studies. To be fair, we are aware of the initiatives of some traditional eye-tracker vendors and other companies to develop a solution for the group eye-tracking use. There are also some open source initiatives 12). Still, as in our case, laboratories tend to develop and maintain their own solutions for practical reasons.

The main source of inadequacy of existing tools is the absence of proper study management features. Especially missing are the features for the centralized remote control and monitoring of the study process. A group study also requires an effective distribution and collection of the required data (stimuli, tasks, logs) to and from the workstations. Finally, a high-level programmatic control of the eye-trackers is seldom available outside of the vendor’s *canned* (closed) tools. Although the eye-trackers do have low-level SDKs, these require a lot of programming effort to be set up for studies. This hampers the integration of external applications, often required in the studies.

We have overcome some of these challenges by building a custom infrastructure for the group eye-tracking laboratory at our institute.

## Infrastructure overview

Our system, the *Group Studies,* was developed and is currently deployed at the User Experience and Interaction Research Center (UXI) at the Slovak University of Technology in Bratislava.

The principal high-level requirements of the *Group Studies* system are:

1. To run the eye-tracking experiments on the individual workstations in the group eye-tracking laboratory (room).
2. To allow a centralized design and scheduling of the experiments.
3. To monitor the experiments centrally.
4. To access the recorded data centrally.

Following these requirements, we designed the *Group Studies* system as a thick client-server application. The system consists of two principal components:

1. *UXR* (UX Research): a web-based management application for administration of the experiments. This application is deployed on a physical server in the laboratory.
2. *UXC* (UX Client): a desktop-based client application, which executes the experiment sessions. This application is deployed on every workstation in the laboratory (PC with an eye-tracker).

Our system works primarily with the Tobii technology but allows the integration with devices from other vendors. It is implemented in C#, utilizes .NET and Windows ecosystem and relies on a fast intranet connection between its elements (10 Gbps in our case). However, since the bulk of the recorded data (screen recordings, eye-tracker logs, etc.) is sent from the individual workstations to the server after the session end, it could be in theory used also in a setup, in which the server and clients do not reside on the same local network.

The system was designed iteratively and incrementally. We base it on our experience with the study organization systems and experimental education platforms, which support group classroom experiments (25, 26). We were also inspired by crowdsourcing systems such as Mechanical Turk and systems for interactive experiment support (21).

Our system distinguishes between the two types of users: (1) the *study owners*, who interact with both UXR and UXC and (2) the *study participants* who interact with the UXC. The study owner role covers the study designers, moderators, and analysts.

**Figure 1:**
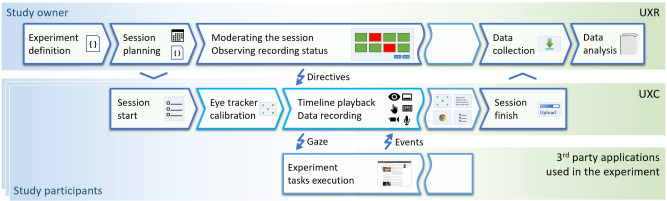
Workflow of a typical experiment conducted in the Group Studies system.

Following is a typical workflow of an experiment in the *Group Studies* (see also Figure 1):

1. The study owner defines the experiment (scenario).
2. The study owner schedules the experiment session(s) using the UXR web interface.
3. During the experiment session, the study participants interact with the UXC (which runs all the necessary steps of the session, e.g., instructions, calibration, stimuli, questionnaires). When necessary, 3^rd^ party applications can exchange events and gaze data with the UXC as well.
4. When the session ends, the UXC uploads all recorded data to the UXR.
5. The recordings are exported from the UXR for further analyses.

*The client application (UXC) is autonomous* to a large extent. The UXC can be run on its own, without the connection to the UXR (the server-side application of our system). This has several advantages. First, when experiments are run in the group session laboratory, the system is less prone to server and network failures. Second, it allows the experiments to be designed and tested anywhere, using only a single machine, even without the eye-tracker (which can be substituted by a mock input).

The experiment is defined using a data structure called the *Session definition*. This structure is stored as a JSON file. It contains various setup parameters and most importantly, the timeline. The timeline is a sequence of stimuli, questionnaires, calibrations, calibration validations and other events, that the participants encounter during the session.

Defining the timeline through JSON files differs from other eye-tracking tools, which usually use graphical interfaces. We chose this approach, because of its:

- *Flexibility*. The experiment owner can write the Session definition JSON anywhere. He/she can then load it directly to an UXC instance (for testing purposes) or distribute it through UXR to all the workstations in the lab (when the real experiment is about to start).
- *Transparency*. The experiment owner can rely solely on the content of the JSON file. There are no "invisible" side effects, as the UXC literally interprets the contents of the timeline.
- *Versionability*. The JSON files can easily be versioned in the source code control tools.
- *Maintainability*. We did not have to write any graphical timeline definition tool, either in the UXR or the UXC (which was a design dilemma on its own). This made future functionality extensions of our system easier.

A downside of using such "programmatic" approach is, of course, the lower accessibility of our system for study owners with a non-technical background. Yet, using the learn-by-example approach, even the non-technical persons can quickly grasp the principles of the JSON session definition, especially when they have the access to a battery of example scenarios.

## System functionality

The following section lists the functionality provided by the *Group Studies* system. We present it component-wise (first UXR, then UXC). In addition, we describe the options available for *Session definition* JSON file, which is defined outside of the both components.

### Functionality of the UXR (server) application

*Create a new project (experiment)*. The experiment owner creates a new experiment in the system, describes it with a name, free-text details, and a session definition file which all newly scheduled sessions will inherit from.

*Schedule the experiment*. The experiment owner plans the experiment in one or multiple sessions. The start and end times of each session must be defined. The session timespans may overlap (we allow the running of sessions in parallel). This allows introduction of some variability in *Session definitions* among participants of the same experiment (for example, the study owner may want to counterbalance the task order). Each scheduled session inherits its *Session definition* from the default definition specified for the project, but it may be modified by the experiment owner in any way, for example to provide alternative stimuli timeline per group of participants.

*Load Session definition*. The experiment owner loads the prepared JSON file with the experiment scenario into the project or an individual session.

*Alter Session definition.* The study owner may alter each Session definition, even for the same project, e.g., to change the tasks or the stimuli between the groups of participants.

*Integrate external applications*. The study owner registers all applications that will interact in real time with the client application (UXC) during the experiment. UXC enables this through the local web API. The interaction may be outbound and/or inbound. The outbound interaction stands for feeding the gaze data to an external application. The inbound interaction represents the case, when the external application feeds arbitrary logs (as JSON objects) into the UXC application, so they are timestamped and can be later retrieved with other recorded data. Such logs may be for example AOI hits occurring in a dynamic environment of the external application resolved by that application itself based on the gaze data retrieved from the UXC (the application should in that case define the AOIs as well; neither UXC nor UXR does not currently allow AOI definition, but it leaves it to the external application or data analyst who may wish to define them manually after the data recording and collection is finished). Another type of inbound interaction is the direct control of the experiment timeline. An external application may force the UXC to advance on the timeline. Or, it may even *insert* a new step into the timeline dynamically, which makes adaptive scenarios possible.

*Remotely observe the state of the workstations in the laboratory*. For each workstation, the study owner can centrally oversee its connection status and the state of its sensors. The information is arranged in a dashboard according to the physical floor plan of the lab. This allows the study owner to quickly track down the problematic workstation and deal with the possible physical issues quickly.

*Start the experiment recording*. The study owner initiates the session on the workstations in the laboratory. Multiple sessions may be started in parallel on different workstations, allowing the study owner to conduct variants of the same experiment. The option for individual manual experiment startup by participants themselves (more suitable in some situations) is also possible in the UXC.

*Retrieve recorded data*. Study owner may retrieve (download) all data recorded in the experiment so far. The data are organized first participant-wise and then source-wise (for each participant, the output of each device is in a separate file).

### Functionality of the UXC (client) application

*Start up the client station*. The workstations in the laboratory are usually started by study owner, not by the participants. After the study owner turns on a workstation PC, the UXC is launched automatically. When running, the application listens for any centrally issued commands and sends updates to the UXR.

**Figure 2:**
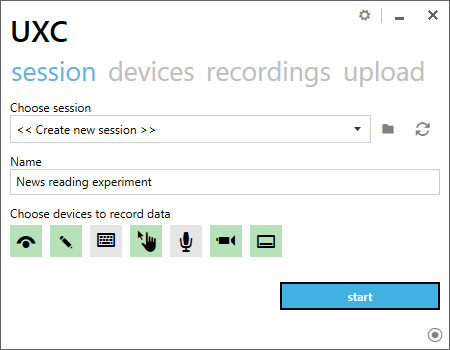
2 UXC application screen. A participant selects a session from a dropdown and starts it using the bottom-right button.

*Start the session recording*. In the experiments, where session does not need to be synchronized, the participants can start the session by themselves. They do so using the main application screen (see Figure 2).

*Calibrate the eye-tracker*. The participant is informed about the need for calibrating the eye-tracker. The calibration is managed by the UXC. It consists of three steps: (1) head-positioning, (2) point animation (default is a 9-point calibration) and (3) calibration result. The result is displayed graphically and can be either accepted or rejected (after which the calibration is restarted). Currently, it is not possible to select the best calibration result from the recent calibrations; this might not even be desirable, since the participant or the eye tracker position (e.g., the screen tilt) might have changed between the different calibration runs. The default calibration behavior (the number of calibration points, etc.) can be overridden in the *Session definition* JSON.

*Validate the calibration*. The participant is informed, that the calibration procedure must be validated. Then, he/she follows the similar procedure as with the regular calibration. This procedure is recommended to be scheduled by the experiment owner at least once somewhere on the experiment timeline. The computation of validation metrics such as accuracy and precision (9) is currently not part of this step and is left to the study owner after the experiment.

*Watch instructions.* An instruction text, centered on a screen, is displayed. The participant proceeds by pressing a “continue” button or after a time limit elapses.

*Fill a questionnaire*. The participant is requested by the system to answer some questions.

*Interact with a stimulus*. The UXC displays the desktop or starts up a program, which the participant is expected to interact with.

*Complete the experiment*. After completing all steps, the recording finishes and the participant is informed about it. The recorded data are transferred to the server where the experiment owner may access them. The upload process can be observed in the UXC (so the participants do not shut down the station too early by accident).

### Session definition JSON schema

Through the *Session definition* JSON, the experiment owner defines a sequence of *steps*. The steps represent the activities in which the participants will be engaged during the experiment session. Also, the study owner defines which devices should be used for the session recording. The complete documentation on the *Session definition* can be found within the UXC GitHub repository^1^.

There are several step types from which a timeline may be composed. Each of these step types can be, to some degree, configured. Each step type can have multiple instances within a single timeline. Each step starts, when the previous one ends. An end of a step can be defined by a hotkey, time limit or an API call (a 3rd party application, used in the experiment, may force a step to end, for example during the *Show desktop* or *Launch program* steps).

The *Group studies* system supports the following timeline step types:

1. *Eye-tracker calibration*. The default 9-point calibration can be overridden with a custom number of calibration points placed on arbitrary locations with an arbitrary order.
2. *Eye-tracker validation*. As with the calibration, the 9-point default can be overridden.
3. *Instructions*. The study owner specifies the instruction text. Optionally, the text size and color and the color of the background can be specified. Also, a continue button may be optionally set up.
4. *Questionnaire*. The study owner defines a set of questions. The questions can be of two types: (1) a free text answer or (2) a pre-defined multi-choice. The answers to a question may be constrained by a regular expression for the text input or by the maximum number of the selected choices. Any question can be marked as required. The text and background style can also be defined for the questionnaires.
5. *Show desktop*. This step serves as means for general recording of the screen. The study owner defines, whether any running applications should be minimized.
6. *Launch program*. This step launches any program available on the workstation machine. The step ends when the program is closed. The study owner specifies a launch command (by specifying path, working directory, etc.). For this command, parameters can be specified. These parameters can take values acquired during the previous session steps (e.g., a user name), which is another option for the scenario adaptation.
7. *Fixation filter*. Usually the last in a session, this step silently executes the event detection algorithm for the eye movement events on the client workstation, e.g., a velocity-based one. The data of eye movement events are transferred to the UXR along with the raw data.

**Figure 3:**
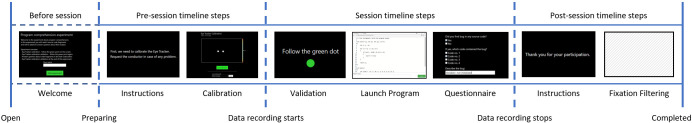
The session timeline is comprised of 3 sub-timelines (pre-session, session and post-session). Each sub-timeline comprises one or more steps. The timelines are defined in a Session definition JSON file.

The timeline consists of three principal subsequent timelines, into which instances of step types can be assigned (see Figure 3 for illustration):

1. *Pre-session timeline*. The steps in this section are executed first and they are not recorded. The eye-tracker calibration step must be placed here. Optionally, the study owner may place other steps here (for example some longer questionnaires that do not require gaze recording).
2. *Session timeline*. The steps in this section are executed after the pre-session timeline and are recorded. In general, all stimuli steps are placed here, along with the respective instructions. The calibration validation steps should be placed here as well.
3. *Post-session timeline*. These steps are executed last and are not recorded. This section can be used for fixation filtering or any other steps which do not require recording.

Apart from defining the timeline steps, the study owner must enumerate, which devices will be used for data recording. Currently, the *Group Studies* supports the following possible data sources:

1. *Eye-tracker*.
2. *External events*.
3. *Keyboard events*.
4. *Mouse events*.
5. *Webcam audio*.
6. *Webcam video*.
7. *Screen recording video*.

Most devices can be recorded automatically without further configuration. The exception are external events, which must be pushed in via local web API by the third-party applications (which the study owners wish to use as stimuli). Also, the quality of audio and video recording can be optionally configured before the recording starts.

## System architecture and physical setup

The *Group Studies* system has two principal components (1) UXR – the web application for experiment management and (2) UXC – the desktop client application for operating the eye-trackers and stimuli. The system also allows the use of (3) external applications, which are often required to serve as stimuli. Figure 7 (appendix) shows interconnection of these system components. Based on the use case, an external application may use any of the interfaces provided by the client application, i.e., push events, read gaze data or even control the experiment timeline.

The role of the UXR (which runs on a web server) is to support the use cases for the study setup and control, as well as retrieving data after the experiment. The UXR also serves for distributing UXC updates. Figure 8 (appendix) shows the main internal components of the management application, built on top of the Microsoft ASP.NET MVC framework and Microsoft SQL Server database.

The client application (UXC) is autonomous during the session execution. The architectural style of the system is a *thick-client*. The client application can receive information (e.g., a session definition) and commands (e.g., a synchronized recording start) from the server (the management application), but apart from that, the *client application manages the session autonomously*. The client application implements the eye-tracker calibration, records the session data (e.g., eye-tracking data, screen recording, user camera, keyboard, and mouse events) and sends them back to the server after the session finishes.

*The autonomous character of the client application is important, because it increases the robustness of the system*, which is thus less prone to server and network failures caused by the bottlenecks.

Figure 9 (appendix) shows the main internal components of the UXC with the *Sessions Control* module for controlling the session recording. The data sources (devices) are controlled automatically by the *Sessions Control* through the *Adapters Control* module. The data source components are adapters, which implement routines required for collecting the specific data types, but which share the same internal interface for the *Adapters Control* module.

The *Eye-tracker* component uses Tobii Pro SDK^2^ library to communicate with a Tobii Pro Eye-tracker device. FFmpeg^3^ is used for recording multimedia: the participant’s screen with the UScreenCapture^4^ software and a webcam available on the workstation. The *Mouse & Keyboard* component records participant’s keystrokes, mouse clicks and movements using the WinAPI provided by the Microsoft Windows operating system. A special data source type is *External Events* which allows external applications to add events recorded during the experiment. During the whole session recording, the experiment timeline is played and gaze data may be accessed by an external application.

When the external applications are going to be used, the study owner, or a developer of the application must implement communication with the UXC local web services, either using REST API or web sockets. The external applications can be either desktop applications or browser-based applications with their own web servers. The external applications communicate with the UXC through the localhost domain. This helps to preserve the overall workstation autonomy. A problem arises if the external application is secured (i.e., uses HTTPS). This can be solved with advanced configuration of the workstation, which we provide details about in the project documentation.

**Figure 4:**
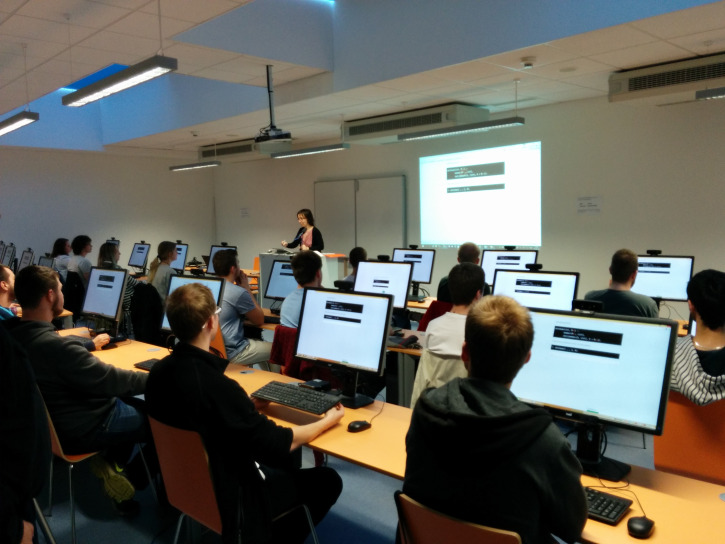
The eye-tracking group lab during an experiment. The layout of the room follows a classroom setup.

Physically, the *Group Studies* system is, with exception of the server, entirely deployed in the room where the group experiments take place. The room can be seen in the Figure 4 during an experiment. 20 workstations are positioned to form a classroom. In our setup, each workstation is equipped with a 60Hz eye-tracker (Tobii Pro X2-60) and a web camera (Creative Senz3D). One additional workstation is dedicated for the study owner and is equipped with a projector. The study owner can use the workstation for controlling the recording of an experiment session. The server side of the system (the management application) runs on a dedicated server, which also hosts the data storage, allowing direct and single-point access to the recorded data.

## Example user study

So far, we have used our infrastructure for several studies. These included studies on cleaning pupillary dilation data from the real-world stimuli lightning effects (10), student attention during an interactive lecture (26) (see also Figure 4), visual search on real websites (7), detecting deception in the questionnaires (20), or the eye-tracking aided crowdsourcing (24).

To demonstrate the use of our *Group Studies* system in the real lab settings, we present a setup from a study for our ongoing research on the program comprehension (27, 11) where participants’ task is to read the source code fragments, understand them and answer the comprehension questions.

### Study motivation

Reading and writing source code is an essential task in software development. The source code reading strategies differ from the typical natural text reading strategies (3). In education, we seek to understand how the novice programmers read, comprehend, and write source code, how they find and repair bugs, and how we can improve their learning process. However, most of the time we only know correctness of their solution, not the process leading to it. The programmer’s visual attention reflects not only the source code itself, but also the programmer’s experience and familiarity with the source code. Research in the empirical software engineering is interested in how the novices differ from the expert programmers and how they can become experts faster.

In these studies, we use eye-tracking to observe how the students in the introductory programming courses solve the programming exercises. During the course’s lab session, the students use an online programming environment, which is integrated with the *Group Studies* system. We collect gaze data and fine-grained interactions with the code editor during the programming session. Then, we are able to reconstruct, analyze and replay the programmer’s activity over time (27). The collected data is used for automatic identification of the program comprehension patterns (e.g. linear scan, retrace declaration, control and data flow tracing) (4). We use these patterns along with the source code-related eye-tracking metrics (22) to train models for predicting the programmer’s performance in the program comprehension tasks, to compare their comprehension strategies, and describe them to the teacher. We explore, whether describing the programmer’s activity in the program comprehension tasks can help the teacher to better identify the student’s misconceptions.

A program source code, although a textual stimulus, differs from natural texts in its structure, semantics, and cognitive processes required for understanding it (4). The previous program comprehension studies with eye-tracking were performed with short code fragments due to the software limitations (3, 15), or tightly coupled with the source code editor (23) which makes them difficult to replicate. The UXI *Group Studies* system enables us to collect data from the program comprehension studies more robustly and efficiently, when compared to the previous works (15). In total, we had 33 participants in this experiment comprising two recording sessions (11).

### The role of the Group Studies system in the studydThe role of the Group Studies system in the study

The UXC client application is used with the Tobii X2-60 eye-trackers to record the gaze data, screen recording, mouse events, and external events. The source code fragments stimuli are presented to the participants on a custom website with the web-based source code editor Monaco^5^. Unlike in the previous studies, the participants can interact with the editor, i.e., scroll the document, move text cursor and select text. If needed, they can also move the window or change its size; we monitor these changes as well. The editor was set to read-only mode for this study, although code changes could be logged as well. All interactions with the editor were translated to the stimulus change logs and pushed as *External events* into the UXC using its local API. The code editor also managed the session through the API for session control.

**Figure 5:**
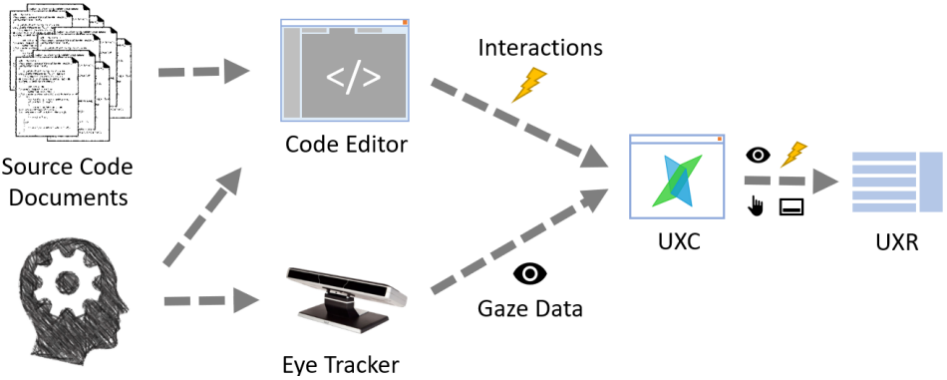
Overview of data recording in the program comprehension study with the UXC. The gaze data, interaction events, mouse events, and screen recording are recorded for all participants. All data is collected in the UXR after the recording

At the beginning of the recording, the eye-tracker calibration was performed, together with the calibration validation before and after the source code reading tasks. Figure 5 outlines the recording part of the study experiment with the UXC. After the recording, all data were collected to the UXR server application.

The experiment sessions took place during the seminars of the introductory procedural programming course at our faculty. The participants were used to work with the web-based source code editors.

**Figure 6:**
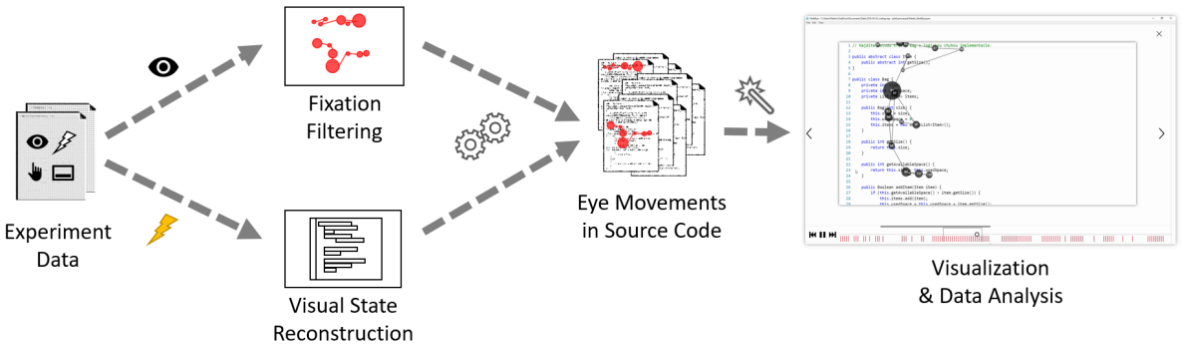
The gaze and interaction data processing from the program comprehension study to reconstruct the visual state of the code editor and fixations relative to the source code documents.

**Figure 7:**
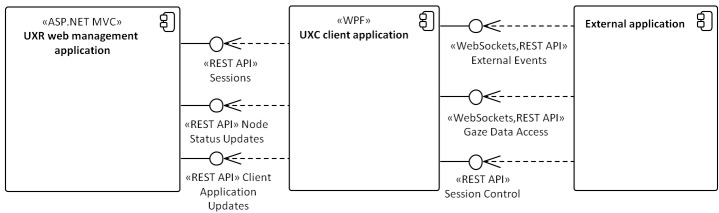
The components overview of the Group Studies system. The web application and the client application are the principal components of the system, with the optional external applications used by the participant during the recording session.

**Figure 8:**
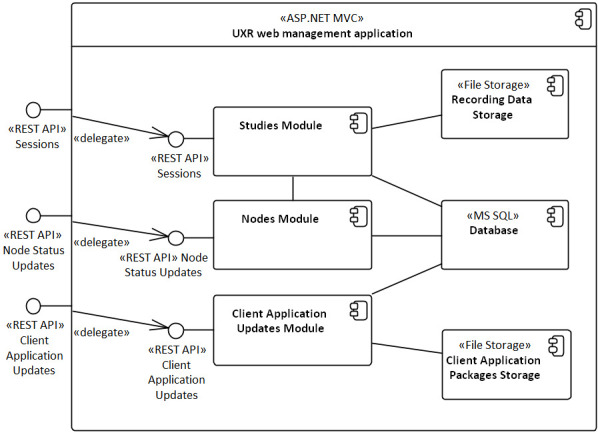
The main internal components of the UXR web application for management of group studies, data storage, access, and distributing client applications in the laboratory.

**Figure 9:**
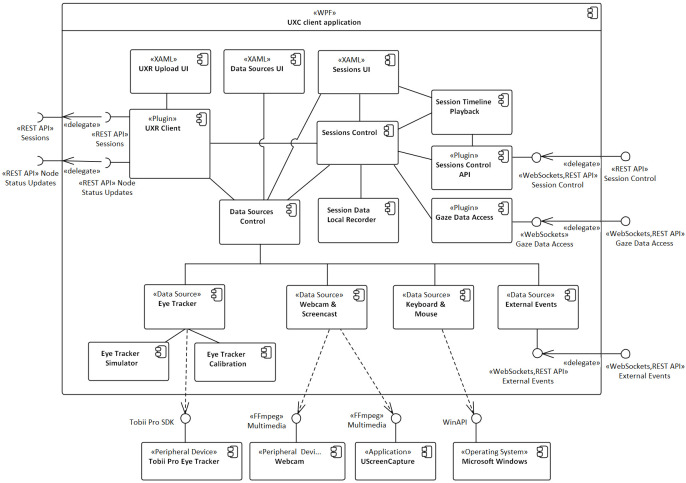
The main internal components of the UXC client application of the Group Studies system. The application automatically records the experiment sessions with multiple devices and allows other external applications to control the recording and access the gaze data.

For the data analysis part Figure 6), we map gaze fixations into positions in the source code documents, while considering where and how each source code fragment was displayed. This mapping is, though, done outside of our system. What is still inside our infrastructure, is the fixation filter we used. It is our implementation of I-VT filter^6^ based on the Tobii whitepaper (17). From the recorded interactions with the source code editor, we reconstruct its visual state for each point in time during the recording, then recalculate fixations to the positions relative to the source code document. Since the source code elements form an AOI hierarchy, such mapping allows us to automatically analyze eye movement data together with AOIs in the source code.

## Source code and documentation

We made the software components of our infrastructure publicly available as source code and documentation. We publish the software in several GitHub repositories:

1. UXC source code^7^
2. UXR source code^8^
3. UXC and UXR dependency libraries^9^

The documentation for the source code is placed within the wiki sections of these repositories.

## Discussion

The *Group Studie*s system is currently best suited for scaling up the eye-tracking experiments, which otherwise have a “single-participant nature”. This means experiments, which do not involve any interaction between the participants (workstations). Many studies are like this and the group eye-tracking simply helps them to be finished faster.

There is, however, an entire line of research dealing with the between-participant interactive eye-tracking scenarios (1, 13, 18, 5). Such scenarios are currently not supported by our system, as there is no direct native support for the data exchange between the workstations.

Despite that, our system does not prevent collaborative scenarios and provides suitable basis for implementing them. By a suitable basis, we mean the local API capabilities of the UXC application. Using this API, an external application can request gaze data from the UXC (UXC can provide the actual scalar or buffered historical data). Therefore, if a collaborative scenario were required to be run on our infrastructure, the data exchange would have to be implemented in the external application.

## Conclusion

The prices of the eye-tracking technologies are steadily dropping and allow larger (hardware) purchases. This allows institutions to furnish more conventional eye-tracking labs, but it also opens the possibility to furnish labs with the group eye-tracking setups. However, running studies in such setups requires special infrastructure to support them.

This paper presented the *Group Studies* system, a software part of a group eye-tracking infrastructure deployed at the User Experience and Interaction Research Center (UXI). The system is based on a thick client-server architecture. It allows flexible preparation of the experiment scenarios and integration of the 3^rd^ party software and is extendable in the future.

We have described the functionality of the system, which supports all required phases of a group eye-tracking experiment. We have also looked at the system from the architectural perspective and shown a high-level overview of its components. This overview serves as a good introduction into the entire implementation of our system, which we made publicly available on GitHub.

The system was primarily designed to support our specific needs in conducting the group eye-tracking studies (at UXI Research Center). It was designed through multiple iterations and evolved over time. Despite that we believe that it can inspire new labs as well. Moreover, researchers can use our code and modify, tailor and deploy this system at their own lab sites.

The system does not support all possible scenarios for the group eye-tracking or the user study designs right now. But also, it does not prevent them. For example, we did not focus it on the collaborative scenarios. Therefore, researchers pursuing this path would be required to put additional effort to use it for these studies. Nevertheless, the ability of our system to integrate external software into the infrastructure would enable such scenarios.

We see several possible directions for the future work. First, we understand that the stimuli timeline structure in its current state may be limiting for certain studies because of its linearity. It is possible to define alternative session timelines when scheduling the session in the UXR or control the stimuli timeline and insert new steps during the recording using the UXC local API from a 3^rd^ party application. However, it is currently not possible to randomize or counterbalance the order of the pre-defined timeline steps, nor is it possible to conditionally select the next step during the recording, possibly based on the results from the previous steps. Another possible feature (and direction for future work), which we identified the need for during our studies, is to validate the eye-tracking data on completion of the *Eye-tracker validation* step during the recording and request the participant to re-calibrate the eye-tracker. Thanks to the design and architecture of the presented system, it will be possible to implement these features in the system in the future.

## Ethics and Conflict of Interest

The authors declare that the contents of the article are in agreement with the ethics described in http://biblio.unibe.ch/portale/elibrary/BOP/jemr/ethics.html and that there is no conflict of interest regarding the publication of this paper.

## Acknowledgements

This article was created with the support of the Slovak Research and Development Agency under the contracts No. APVV-15-0508 and APVV-17-0267, the Scientific Grant Agency of the Slovak Republic, grant No. VG 1/0646/15, the Ministry of Education, Science, Research and Sport of the Slovak Republic within the Research and Development Operational Programme for the project “University Science Park of STU Bratislava”, ITMS 26240220084, co-funded by the ERDF and the project “Development of research infrastructure STU”, project no. 003STU-2-3/2016 by the Ministry of Education, Science, Research and Sport of the Slovak Republic.

We wish to thank Tobii Pro for fruitful collaboration with setting up and maintaining our lab infrastructure.

## Footnotes

https://github.com/uxifiit/UXC/wiki/Stimuli-Timeline-Definition https://github.com/uxifiit/UXC/wiki/Session-Recording-Definition

https://www.tobiipro.com/product-listing/tobii-pro-sdk/

https://www.ffmpeg.org/

http://umediaserver.net/components/index.html

https://microsoft.github.io/monaco-editor/index.html

https://github.com/uxifiit/UXI.GazeToolkit/

https://github.com/uxifiit/UXC

https://github.com/uxifiit/UXR

https://github.com/uxifiit/UXI.Libs
